# Physicochemical and Sorptive Properties of a Phosphorylated Mercerized Cotton Fabric

**DOI:** 10.3390/polym13213756

**Published:** 2021-10-30

**Authors:** Roman Solovov, Anfisa Perevoznikova, Alexander Seliverstov, Alexey Shapagin, Alexandr Fedoseev, Vitaly Milyutin, Boris Ershov

**Affiliations:** Frumkin Institute of Physical Chemistry and Electrochemistry of the Russian Academy of Sciences, 40 Obruchev Street, 117342 Moscow, Russia; perevoznikova1723@mail.ru (A.P.); alex_sel@bk.ru (A.S.); shapagin@mail.ru (A.S.); a.fedosseev@gmail.com (A.F.); vmilyutin@mail.ru (V.M.); ershov@ipc.rssi.ru (B.E.)

**Keywords:** phosphorylated cotton cellulose fabric, mercerisation, sorption, static exchange capacity, copper, americium, uranium, plutonium

## Abstract

A process of phosphorylation for a mercerized cotton kersey fabric was investigated. After wet oxidation, the phosphorus content in each sample was determined by spectrophotometric analysis. The range was 0.179 to 0.950 mmol g^–1^. A significant decrease in the tensile strength of samples resulted from an increase of phosphoric acid concentration in the phosphorylating solution. The mercerization has a positive impact on the process of phosphorylation, as the phosphorus content was found to be three times higher in the samples that underwent mercerization. The sorption properties of phosphorylated cotton fabric were studied using the Cu^2+^ sorption process as a reference. The value of the static exchange capacity for the phosphorylated fabric was determined to reach its maximum when the concentration of the H_3_PO_4_ in the phosphorylating solution was 1.40 M, and was found to be 1.48 ± 0.11 mmol g^–1^ with the phosphorus content equal to 0.898 ± 0.090 mmol g^–1^. The sorption of Cu^2+^ by a single phosphorus-containing group occurred for samples with phosphorus content not exceeding 0.80 mmol g^–1^. The preliminary studies of micro-quantities of ^241^Am, ^233^U, and ^239^Pu radionuclide sorption from aqueous solutions with phosphorylated textile demonstrated the high efficiency.

## 1. Introduction

Cellulose is one of the most common natural linear syndiotactic homopolymers. Cellulose is formed by glucopyranose units linked through β-(1,4)-glycosidic bonds. Due to the free hydroxyl groups being linked with a pyranose ring, it is possible to carry out chemical reactions typical for primary and secondary alcohols. Interactions that do not lead to the destruction of the pyranose ring and retain the original structure of the polymer are mainly used, such as the formation of ethers or esters, carboxylation, sulphonation, phosphorylation, and amination [1,2,3,4,5]. When modifying cellulose by introducing new functional groups, one can achieve a cellulose-based compound with new properties and many possible applications. For example, modified cellulose materials are widely used in water filtration technologies, heavy metal sorption processes, and the development of new non-biodegradable products for medicine and fire-retardant materials. [6,7,8,9,10,11,12,13,14]. A lot of research focuses on phosphorylated cellulose. First, phosphorus-containing groups are introduced and then linked through covalent bonds to the cellulose molecule by treating it with phosphorus-containing compounds, such as P_2_O_5_, PCl_5_, PCl_3_, POCl_3_, and H_3_PO_4_ [3,5,14,15,16,17]. However, the studies focusing on the phosphorylation process of preliminary mercerized cotton fabric are relatively sparse. Preliminary mercerization of cellulose significantly increases its chemical activity in the etherification processes and improves sample wettability, associated with the sliding effect and smoothness of the cellulose fibers, and reduces the edge angle of wetting. Mercerization improves the absorptive properties of the fabric and makes treatment with various solutions easier.

After our samples were treated with NaOH followed by washing, the partial process of protonation-deprotonation occurred, resulting in surface hydrophilization with an increase in the hydrophilic–lipophilic balance of the fabric. The phosphorylated mercerized cotton materials can be used as active and easily impregnable sorbents in filters, bases for applying defined compositions to a material’s surface, and disinfecting agents [18,19]. The development of special flushable compositions allows reusing the fabric after it depletes its disinfecting abilities.

We report new results of an elaborate study of phosphorylated mercerized cotton cellulose properties in the present work.

## 2. Materials and Methods

### 2.1. Cotton Cellulose Fabric Treatment

Commercially available mercerized cotton cellulose fabric (Moscow, Russia) with the relative density of 390 ± 9 g/m^2^, plain-weave texture, fiber diameter 250 ± 30 µm, and gage 700 ± 50 µm was used. Mercerization of cotton fabric in production was carried out by short-term processing of the fabric with a concentrated NaOH (BioXtra, ≥98% (acidimetric), pellets (anhydrous)) solution under tension in the cold, followed by rinsing with hot and cold water.

The dried fabric samples containing no more than 10% of moisture were treated with 2.80 mol L^–1^ urea solution (Merck, GR for analysis ACS, Reag. Ph Eur) and phosphoric acid (ACS reagent, ≥85 wt. % in H_2_O) (the concentration was in the range of 0.200 to 2.403 mol L^–1^) at 80 °C for 1 h (the ratio of «sample» to «liquid» was 1:10). The treated samples were each squeezed to a mass two times greater than the dry sample mass and dried at 70 °C for at least 5 h. After drying, the samples were heated at 145–150 °C for 1.5 h. After the phosphorylation process, the samples were washed three times with hot and cold distilled water and were treated with the 0.10 mol L^–1^ HCl (ACS reagent, 37%) solution at room temperature for 1 h. Then, the samples were washed three times with cold distilled water again and were dried at 70 °C for at least 5 h.

### 2.2. Phosphorus Content Determination

The phosphorus content in cotton fabric before and after phosphorylation was determined by the spectrophotometric method based on the color intensity of the Sn(II) reduced phosphorus molybdenum complex. To convert the phosphorus in the samples to phosphate ions, approximately 500 mg of each sample was treated with a 25 mL hot mixture consisting of 3 volumes of concentrated sulfuric acid (ACS reagent, 95.0–98.0%) and 1 volume of 60% hydrogen peroxide (Dzerzhinsk, Nizhny Novgorod region). When placed in the solution, the samples dissolved completely, and the release of gases accompanied the reaction. After that, the solution was heated at 175 °C for 1 h to decompose the unreacted excess of hydrogen peroxide and then transferred into a flask and diluted with distilled water. This kind of treatment and preparation for analysis ensured that all the phosphorus in the phosphorylated sample transformed into phosphoric acid, which allowed determining its content later on. All solutions used and distilled water were thermostabilized beforehand in a water thermostat to 25 °C. Then, 500 mL of the sample was transferred to a 50 mL flask and diluted with distilled water to volume. After thorough mixing, 2 mL of 2% sulfurous ammonium molybdate (NH_4_)_2_MoO_4_ (Sigma Aldrich, 99.98% trace metals basis) was added, and 0.200 mL of 3.5% SnCl_2_ (Sigma Aldrich, reagent grade, 98%) was added after 5 min. The solution was mixed at 25 °C for 15 min. The optical density of the blue phosphor-molybdenum-reduced complex was detected at a wavelength of 700 nm (a 5.00 cm thick cell was used). The experimental molar absorption coefficient for this complex was found to be equal to ε_700 nm_ = (2.485 ± 0.023) × 10^4^ cm^−1^ L mol^−1^. According to the previously obtained calibration graph, the phosphorus content in fabric was determined as mmol g^–1^ or mass percentages.

### 2.3. Infrared Spectroscopy

The samples were analyzed on the FTIR (FT-02, INFRALUM, Lumex, St. Petersburg, Russia) spectrometer in the range of 400–4000 cm^−1^. To do so, 200–300 mg of the fabric samples were thoroughly ground with twice recrystallized KBr (Sigma Aldrich, FT-IR grade, ≥99% trace metals basis) to obtain a homogeneous mixture with a 0.5% cellulose material mass fraction.

### 2.4. Acid–Base Titration

The acid–base titration of the phosphorylated mercerized cotton cellulose was carried out through potentiometric titration with 0.100 mol L^–1^ NaOH using the pH meter («ECOTEST 120», Econix, Moscow, Russia) (glass electrode combined with silver chloride reference electrode). 

### 2.5. Tensile Strength

Tensile testing was performed on a Zwick Roell Z010 (Zwick/Roell, Germany) setup. The width of the sample was shown to be 20 mm, thickness—0.70 mm, preload value—0.10 N, stretching speed—20 mm/min. The standard maximum of the breaking force was measured until at least 5 reproducible results were obtained. The results were processed using t-test statistics.

### 2.6. Sorption Characteristics

The sorption characteristics of the samples were determined by studying the sorption process of macro-quantities of Cu^2+^ and UO_2_^2+^ and micro-quantities of ^241^Am, ^233^U, and ^239^Pu radionuclides. 

#### Sorption of Cu^2+^ and UO_2_^2+^

The sorption properties of the samples were studied on a model of Cu^2+^ and UO_2_^2+^ sorption from CuSO_4_ (anhydrous, powder, ≥99.99% trace metals basis) (the concentration was in the range of 1–200 mmol L^–1^) and 20 mmol L^–1^ UO_2_(NO_3_)_2_ (99.9%, Russia) solutions. About 0.5 g of the sample was soaked in a pH 3.0 solution of sulfuric acid at 20 °C for 5 days while stirring (the ratio of «sample» to «liquid» was 1:50). The pH changes after sorption were detected to be no more than 0.5. The Cu^2+^ concentration before and after soaking the sample in the solution was determined spectrophotometrically with the Cary Varian 100 device based on the ammonia complex color intensity at λ = 630 nm in the presence of a 1000-fold excess of the ligand (ε_630_ = 69.5 ± 0.5 cm^–1^ L mol^–1^). 

The UO_2_^2+^ concentration in the solution was determined spectrophotometrically on the Shimadzu UV 3100 (Shimadzu Corporation, Japan) device based on the color intensity at λ = 420 nm. The values of the static capacity were calculated using the following equation:

(1) $$SEC=\frac{\left(C_{0}\;-\;C_{eq}\right){\;\cdot \;V}_{s}}{m_{t}}$$

where *SEC*—static exchange capacity for copper or uranyl ions, mmol g^–1^; *C_0_*—initial concentration of copper or uranyl ions in the solution, mmol L^–1^; *C_eq_*—equilibrium concentration of copper or uranyl ions in the solution, mmol L^–1^; *V_s_*—the volume of the solution, L; *m_t_*—sample mass, g.

### 2.7. Sorption of Radionuclides ^241^Am, ^233^U, and ^239^Pu 

Caution! ^241^Am, ^233^U, and ^239^Pu are highly radioactive isotopes and require specially equipped facilities for safe work. 

About 0.1 g of the fabric sample (precision of weighing—±0.0001 g) was soaked in a prepared solution at 20 °C for 48 h while stirring (the ratio of «sample» to «liquid» was 1:200). The specific activity of ^241^Am, ^233^U, or ^239^Pu radionuclides was determined using the filtrate above the solid phase.

The sorption of ^241^Am and ^233^U was tested on Moscow tap water with the following composition: mg L^–1^: Na^+^, 6–8; K^+^, 4–5; Mg^2+^, 15–17; Ca^2+^, 52–56; Cl^–^, 6–8; SO_4_^2–^, 36–38; HCO_3_^–^, 200–205; total mineral content, 310–330; hardness, 3.6–3.8 mmol L^–1^; pH = 7.3–7.8. The appropriate amount of the corresponding radionuclide (about 10^5^ Bq L^–1^ ≈ 4.3 mg L^–1 233^U) was added to the liquid phase and kept for 120 h to establish hydrolytic equilibrium between the radioactive and inactive components of the solution. The solution for investigating plutonium sorption contained 1.0 mol L^–1^ HNO_3_ and about 10^6^ Bq L^–1 239^Pu and was prepared by dissolving 1.0 mg [N(CH_3_)_4_]_2_Pu(NO_3_)_6_ in an aliquot of 3.0 mol L^–1^ HNO_3_ and diluting it with water. The fabric samples were added to the solution and soaked in it. After the sorption equilibrium was reached, the partition coefficient was calculated with the following equation:

(2) $$K_{d}=\frac{A_{0}{\;-\;A}_{eq}}{A_{eq}}\;\cdot \;\frac{V_{s}}{m_{t}}$$

where *K_d_*—partition coefficient, ml g^–1^; *A_0_* and *A_eq_*—initial and equilibrium specific activity of radionuclides, Bq L^–1^; *V_S_*—solution volume, ml; *m_t_*—fabric mass, g.

The specific activity of ^241^Am in solutions was determined by a radiometric method from the intensity of 59 keV γ-line using an SKS-50M spectrometric complex («Green Star Technologies», Australia). The specific activities of ^233^U and ^239^Pu in solutions were determined by an alpha-spectrometric method from the intensities of 4.9 and 5.2 MeV lines, respectively, using a low-background semiconductor alpha spectrometer ALPHA-ARIA («ORTEC», USA).

## 3. Results and Discussion

**Figure 1:**
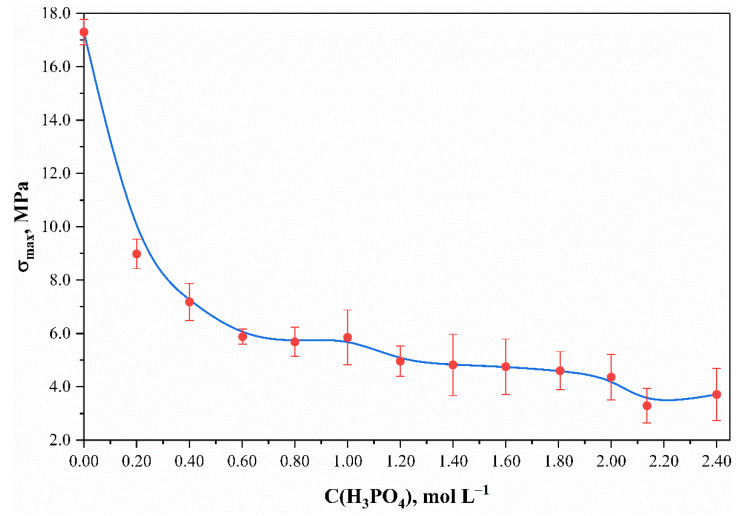
Effect of phosphoric acid content on the standard maximum tensile strength for phosphorylated cotton fabric samples.

Both external and structural changes were the results of the phosphorylation process of preliminary mercerized cotton cellulose. The sample had a yellowish color after being heated up to 145 °C, the intensity of which subsequently faded after being washed in hot and cold distilled water. According to the tensile strength test results, the heat treatment did not reduce the sample strength characteristics during phosphorylation in the absence of H_3_PO_4_. The standard maximum tensile strengths for untreated and phosphorylated samples were observed to be equal to 17.2 ± 0.5 and 17.9 ± 0.6 MPa. The difference between the statistical error margins indicates that there were no changes in the molecular and supramolecular structure after both mercerization and heat treatment of the cotton fabric. However, even a low concentration (0.20 mol L^–1^) of H_3_PO_4_ caused a decrease in the sample’s strength characteristics by almost two times (Figure 1; apparently, it was caused by the changes in the structures of the molecules and their partial destruction, which seemed to increase when increasing the phosphoric acid concentration in the phosphorylating solution). However, it should be noticed that the full destruction of the sample was not observed at H_3_PO_4_ concentration up to 2.403 mol L^–1^.

The results of phosphorus content determination are shown in Table 1.

Table 1 shows a noticeable increase in phosphorus content in the samples, which corresponds to the increased phosphoric acid concentration in the phosphorylating solution. The number of phosphorus-containing groups per 1 g of the sample increased from 0.179 to 0.950 mmol, while the phosphoric acid concentration increased from 0.201 to 2.00 mol L^–1^. However, a further increase in the acid concentration did not increase phosphorus content, which was constant within the margin of statistical error.

**Figure 2:**
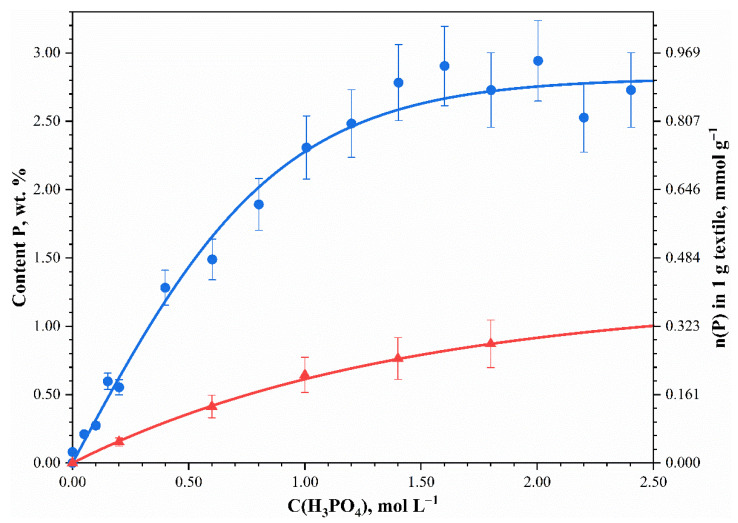
The phosphorus content (wt.% and mol in 1 g textile) in the phosphorylated mercerized (circles) and unmercerized (triangles) fabrics versus phosphoric acid concentration in the phosphorylating solution.

For comparison, the unmercerized cotton fabric was phosphorylated, and phosphorus content in it was determined. Unmercerized cotton fabric is less hydrophilic and is more difficult to treat with aqueous solutions. Figure 2 shows the phosphorus content in the phosphorylated mercerized and unmercerized fabrics against the phosphoric acid concentration in the phosphorylating solution. The phosphorus content increased with the increase in the phosphoric acid concentration, for both the mercerized and the unmercerized fabric. However, phosphorus content increased nonlinearly to about 3 wt.% in the mercerized fabric and 1 wt.% in the unmercerized fabric. The phosphorus content in the mercerized sample was found to be three times greater than in the unmercerized one.

**Figure 3:**
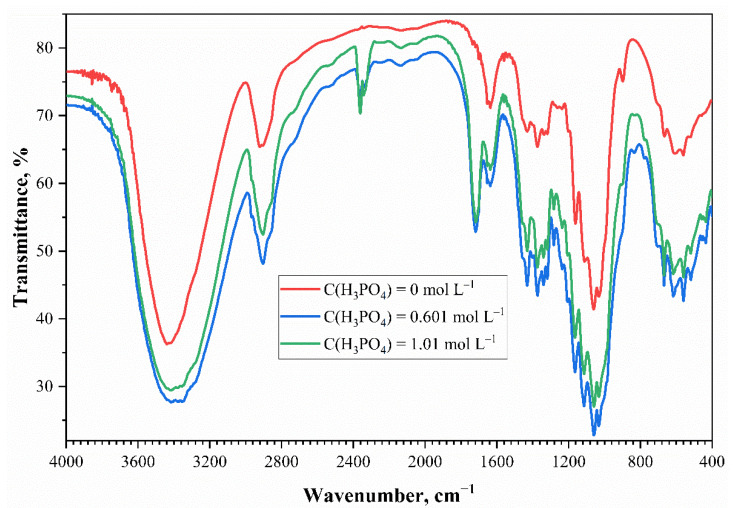
The IR spectrum of the mercerized cotton cellulose and the phosphorylated-cotton, cellulose-treated solutions with different phosphoric acid concentrations.

The IR spectroscopy results of the mercerized cotton cellulose samples are shown in Figure 3.

According to Suflet and Lehtonen [2,3], a mercerized cotton cellulose sample has specific IR wavenumbers: O–H groups stretching vibrations at 3400–3500 cm^–1^, CH_2_-groups at 2800–2900 cm^–1^, and C–O–C elements of the glycosidic bonds at 1166 and 1115 cm^–1^. Several additional bands in the IR spectrum of the phosphorylated cotton cellulose were obtained. The absorption band at 1720 cm^–1^ indicates the presence of the C=O groups that were probably formed after the destruction of the pyranose ring and the partial oxidation of cellulose at high temperatures and formation of carbamates [20,21,22,23]. Presumably, it means partial oxidation of the sample. The absorption band at 2370 cm^–1^ belongs to the P–H stretching vibrations, the absorption band at 1210 cm^–1^ to the P=O stretching vibrations, and the absorption band at 830 cm^–1^ to the P–O–C stretching vibrations. The absence of additional bands in the 2800–2900 cm^–1^ region and the shoulder at 920–1000 cm^–1^, coming from the P–O–H valence vibrations, indicate the absence of the said functional groups. This was also confirmed by the inability to perform acid–base titration on phosphorylated cotton cellulose. The potentiometric titration curve does not show the expected jump when titrating a crushed sample with a KOH solution. This fact proves that there were no functional phosphorus-containing groups with hydrogen that could be substituted.

**Figure 4:**
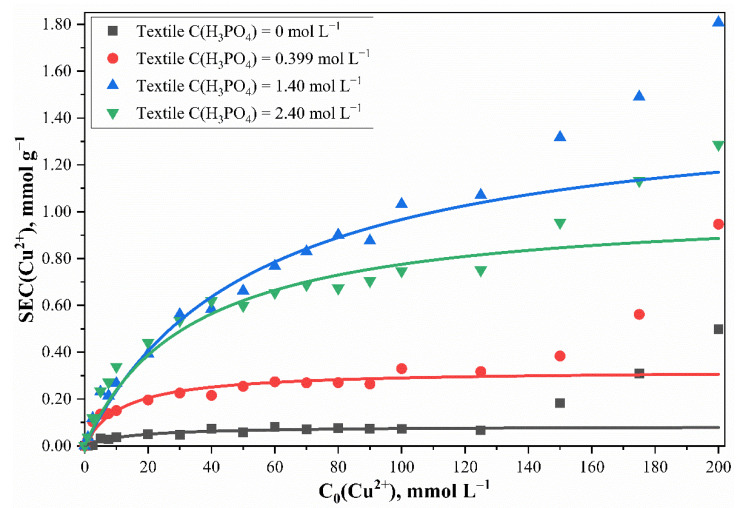
Cu^2+^ sorption isotherms of cotton cellulose samples phosphorylated with phosphoric acid solutions of different concentrations. Points—experimental data; lines—calculated values according to the Langmuir equation. The temperature of the solutions during sorption experiments was t = 20 °C.

The modified phosphorylated cotton cellulose can be used as a toxic heavy metal sorbent [24,25,26]. We tested the sorption properties of the obtained phosphorylated cotton cellulose samples using aqueous solutions of copper as an example, since it can be assumed that similar behavior under these conditions would be demonstrated by other divalent metals, such as nickel, cobalt, iron, lead, and strontium. The sorption properties of the phosphorylated cotton cellulose were studied by using Cu^2+^ sorptive properties in solutions with different concentrations as a reference (pH = 3.0, t = 20 °C). The obtained data demonstrate a significant increase in the sorption capacity in the presence of the phosphorus-containing groups. Furthermore, the shapes of the adsorption isotherms and their non-linearity indicate a complicated mechanism of the adsorption process. Presumably, chemosorption occurs on the surface due to complexation along with the Cu^2+^ physical adsorption. The obtained data were described by the Langmuir monomolecular adsorption model (Figure 4). The plateau corresponds to the saturation of sorption centers and formation of a monomolecular layer according to the equation:

(3) $${SEC=SEC}_{\infty }\;\cdot \;\frac{K\;\cdot \;C\left({Cu}^{2+}\right)}{1+K\;\cdot \;C\left({Cu}^{2+}\right)}$$

where *SEC*—static exchange capacity, mmol g^–1^; *SEC_∞_*—maximum static exchange capacity, mmol g^–1^; *K*—exchange equilibrium constant—the ratio of sorption rate constants to desorption rate constants, L mmol^–1^; *C*(*Cu^2+^*)—copper ions concentration in the solution, mmol L^–1^.

However, a further increase in *SEC* along with an increase of the sorbed Cu^2+^ concentration indicates the polymolecular nature of sorption. In this case, the rise of the sorption isotherm at more than 125 mmol L^–1^ of Cu^2+^ is explained by the sorption of additional copper amounts and the appearance of the second and subsequent adsorption layers, and the occurrence of chemosorption.

**Figure 5:**
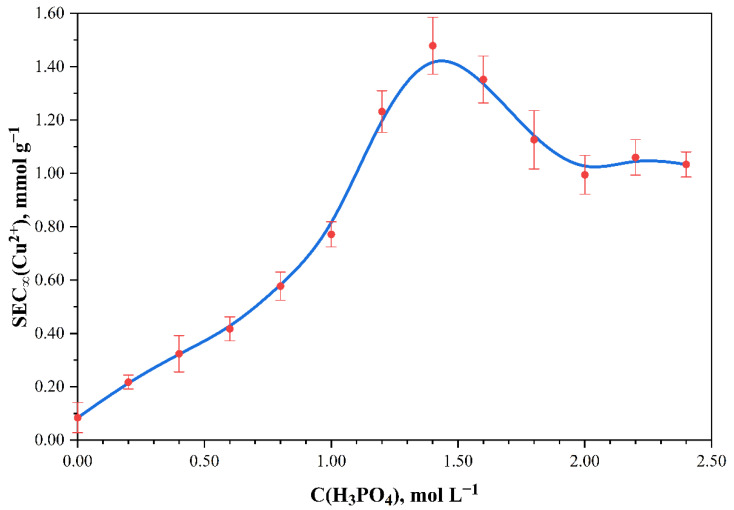
Effect of phosphoric acid concentration on the maximum static exchange capacity *SEC_∞_* (t = 20 °C) of phosphorylated cotton cellulose samples related to Cu^2+^.

Thereby it is appropriate to compare the maximum static exchange capacity of the monomolecular layers *SEC_∞_* of different phosphorylated cotton cellulose samples (Figure 5).

**Figure 6:**
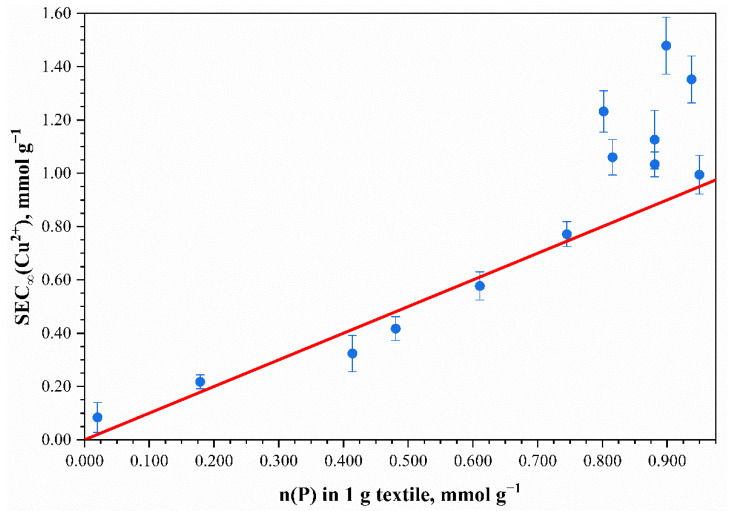
Effect of phosphorus content in mercerized phosphorylated cotton cellulose on the maximum static exchange capacity *SEC_∞_* (t = 20 °C) related to Cu^2+^. Points—experimental data; the line corresponds to the *n*(Cu^2+^):*n*(P) = 1:1 ratio.

It is clear that sample phosphorylation, even with low concentrations of phosphoric acid, led to an increase in the maximum sorption capacity. The results confirm that the phosphate groups were added to the monomer units of the cotton cellulose. In addition, the initial part of the curve shows an increase in the Cu^2+^ sorption capacity and an increase in the concentration of H_3_PO_4_ in the phosphorylating solution. The *SEC_∞_* maximum (1.48 ± 0.11 mmol g^–1^) was observed with 1.40 mol L^–1^ of H_3_PO_4_. The maximum sorption capacity did not change up to 1.80 mol L^–1^ of H_3_PO_4_ within the margin of error. A further increase in H_3_PO_4_ concentration caused a significant decrease in *SEC_∞_*. Furthermore, considering a dependence between amount of Cu^2+^ absorbed and phosphorus content, it can be concluded that a linear correlation exists (Figure 6). Cu^2+^ coordinated in the ratio of *n*(Cu^2+^):*n*(P) = 1:1 with no more than 0.80 mmol g^–1^ of phosphorus content. The exchange equilibrium constant for the samples with phosphorus content no more than 0.80 mmol g^–1^ was found to be 0.0803 ± 0.0087 L mmol^–1^. For the samples with the phosphorus content of 0.80 mmol g^–1^ and higher, the exchange constant was equal to 0.0331 ± 0.0077 L mmol^–1^. When studying the sorption properties of the phosphorylated cotton fabric with phosphorus content exceeding 0.80 mmol g^–1^, it was discovered that Cu^2+^ sorption increased, and the *n*(Cu^2+^):*n* (P) = 1:1 ratio was no longer true. This can be explained by the bidenticity of phosphorus-containing groups and their ability to coordinate more metal ions than in the case of a 1:1 ratio. This proportionality indicates a complexation reaction and the chemical nature of the sorption.

Sorption of uranyl ions, americium(III), and plutonium(IV) was studied, since they are usually present in radioactive waste and are the most radiotoxic elements, primarily due to the long half-lives of their main isotopes present in radioactive waste. In addition, the obtained data can help to predict the general sorption behavior for all f-elements in their specified oxidation states.

**Figure 7:**
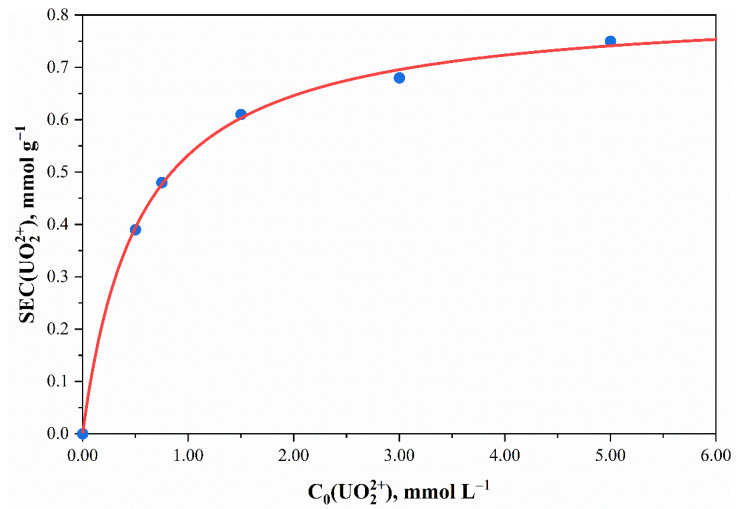
UO_2_^2+^ sorption isotherm of cotton cellulose samples phosphorylated with 0.802 mol L^–1^ H_3_PO_4_ solution (phosphorus content—1.89 ± 0.19 wt.%; *n*(P) = 0.611 ± 0.061 mmol g^–1^). Points—experimental data; lines—calculated values in accordance with the Langmuir equation. The temperature of the solutions during sorption experiments was t = 20 °C.

The isotherm of uranyl ions sorption was obtained from a sample phosphorylated with 0.802 mol L^–1^ H_3_PO_4_ solution (phosphorus content—1.89 ± 0.19 wt.%; *n*(P) = 0.611 ± 0.061 mmol g^–1^) (Figure 7).

The obtained experimental data are well described by the Langmuir equation. The maximum static exchange capacity (*SEC_∞_*) and the exchange equilibrium constant (K) for the uranyl ions sorption by the phosphorylated fabric sample were 0.822 ± 0.014 mmol g^–1^ and 1.844 ± 0.11 L mmol^–1^. The high values of the exchange constants and the convex shape of the sorption isotherms indicate that the uranyl ions sorption by phosphorylated cellulose samples is highly selective.

**Figure 8:**
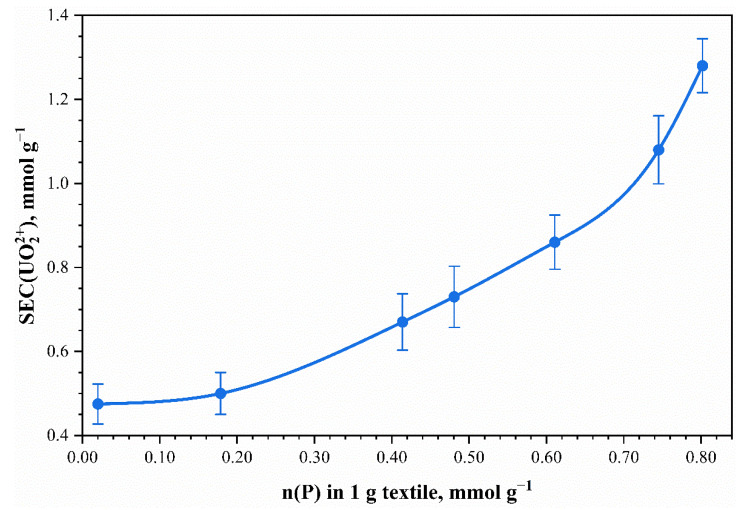
Effect of phosphorus content in mercerized phosphorylated cotton cellulose on the static exchange capacity *SEC* from 20 mmol L^–1^ UO_2_^2+^ solutions. Points—experimental data; the line corresponds to the ratio *n*(Cu^2+^):*n*(P) = 1:1. The temperature of the solutions during sorption experiments was t = 20 °C.

The dependence of the static exchange capacity UO_2_^2+^ on the phosphorus content in the fabric samples is shown in Figure 8.

The presented results (Figure 8) show that an increase of phosphorus content in the samples increased the capacity of the uranyl ions. This indicates an increase in the number of ionogenic groups in the composition of sorbents when using more concentrated phosphoric acid solutions in the phosphorylation process. Higher absolute values of static exchange capacity for uranyl ions than static exchange capacity for copper are apparently associated with the formation of stronger uranium compounds with phosphate groups on cellulose.

**Figure 9:**
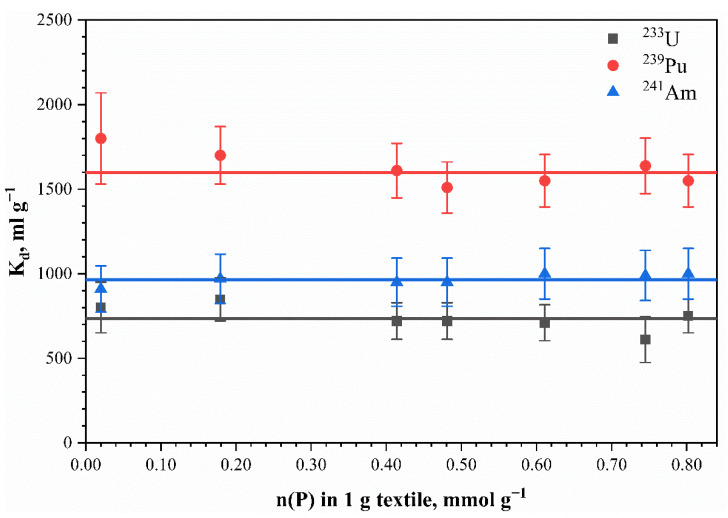
Effects of phosphorus content in mercerized phosphorylated cotton cellulose on the partition coefficient for different radionuclides. Points—experimental data; lines correspond to the partition coefficients. The sorption of ^241^Am and ^233^U was tested on Moscow tap water; sorption of ^239^Pu—with 1.0 mol L^–1^ HNO_3_ solution.

The distribution coefficient’s (*K_d_*) dependence for ^241^Am, ^233^U, and ^239^Pu radionuclides on the phosphorus content in the phosphorylated cellulose samples is shown in Figure 9.

The results presented in Figure 9 demonstrate no statistically significant change in ^241^Am, ^233^U, and ^239^Pu radionuclide sorption in relation to increasing the phosphorus content in the fabric sorbents. The average partition coefficient (*K_d_*) values for radionuclides were 965 ± 13, 1600 ± 32, and 734 ± 25 mL g^–1^, respectively. In the case of ^241^Am and ^233^U, the observed effect can be explained with their presence in tap water in hydrolyzed forms, i.e., corresponding hydroxides or radiocolloids. Sorption of these forms of radionuclides occurs due to physical adsorption on the sorbent surface and does not depend on the degree of phosphorylation of the cellulose. The absence of the effect of the phosphorus content on the sorption of ^239^Pu may have been due to the high complexing ability of phosphate groups for Pu^4+^ ions. In this case, almost complete sorption (more than 99%) of microquantities of plutonium from the acidic solution occurred despite the change in ionogenic groups’ concentrations in the sorbent. It should be noted that physical adsorption of hydrolyzed and polymeric forms of plutonium (which can be formed in 1.0 mol L^–1^ HNO_3_) can occur.

The research results demonstrating such a significant static exchange capacity of uranyl ions provide the possibility of using pre-mercerized phosphorylated cotton fabric as a sorbent of other radioactive actinides. This promising sorbent with pre-combustion allows the compaction of ash into cement matrixes and the disposal of radioactive waste.

## 4. Conclusions

Mercerized cotton fabric is a cheap, commercially available raw material used in the production of woven materials for various purposes. Therefore, it presents the possibility to create modified materials with other functional groups in order to achieve desired physicochemical properties. Phosphorylation of cotton cellulose fabric reduces its strength, and high concentrations of phosphoric acid in the phosphorylating solution (more than 2.40 mol L^−1^) lead to significant destruction of the fabric, which deprives it of advantages in application and regeneration over powdered cellulose.

The study of mercerized cotton cellulose samples by IR spectroscopy and their potentiometric titration showed that the P=O and C–O–P functional groups are present in the structure of the modified cellulose. The absence of a significant increase in the Pu^4+^ sorption with an increase in phosphorus content may indicate the absence of a hydroxyl group on the phosphorus atom. This behavior is observed in mono- and dialkyl phosphoric acids that form extremely strong complexes with tetravalent f-elements ions. Our sorption study of microquantities of actinides in different oxidation states from tap water shows that a noticeable amount of bicarbonate ions levels out the difference in the sorption behavior of the studied elements. It allows us to assume that significant contributions by physical sorption and the formation of radiocolloids occurred. In the case of Pu^4+^ sorption, the typical pattern of the tetravalent f-elements’ ions’ interaction with trialkyl phosphates was observed. That confirms the assumption that phosphoryl groups were present in the modified cellulose in a form close to trialkyl phosphate.

Data analysis on the static exchange capacity of pre-mercerized phosphorylated cellulose indicates a complex polymolecular adsorption mechanism, which can be described by the Langmuir equation. It was found that coordination of a copper ion occurs by a single phosphorus-containing group (*n*(Cu^2+^):*n*(P) = 1:1) with 0.80 mmol g^–1^ of phosphorus in the sample.

The mercerization process is also simple and easily feasible at the place of production. Hence, the obtained results are of interest for developers of new woven materials with high phosphorus content and the ability to absorb heavy and carcinogenic metals. In addition, the discovered high sorptive ability of the studied fabric to uranyl ion opens up the possibility of burial of radioactive actinides by ash cementation after complete combustion of the spent sorbent.

The obtained results make it possible to implement phosphorylated cellulose as a material suitable for collecting and removing small volumes of technological solutions containing valuable or hazardous metals for recuperation or waste disposal.

## Figures and Tables

**Table 1:** Results of the phosphorus content determination in the cotton cellulose samples before and after phosphorylation, obtained by the spectrophotometric method based on the color intensity of the reduced phosphorus molybdenum complex.

№	C(H_3_PO_4_), mol L^–1^	Content P, wt.%	*n*(P) in 1 g Textile, mmol g^–1^
1	0	0.062 ± 0.006	0.0201 ± 0.0020
2	0.201	0.55 ± 0.06	0.179 ± 0.018
3	0.399	1.28 ± 0.13	0.414 ± 0.041
4	0.601	1.49 ± 0.15	0.481 ± 0.048
5	0.802	1.89 ± 0.19	0.611 ± 0.061
6	1.01	2.31 ± 0.23	0.745 ± 0.075
7	1.20	2.48 ± 0.25	0.802 ± 0.080
8	1.40	2.78 ± 0.28	0.898 ± 0.090
9	1.60	2.90 ± 0.29	0.938 ± 0.094
10	1.80	2.73 ± 0.27	0.881 ± 0.088
11	2.00	2.94 ± 0.29	0.950 ± 0.095
12	2.20	2.53 ± 0.25	0.816 ± 0.082
13	2.40	2.73 ± 0.27	0.881 ± 0.088

## Data Availability

Not applicable.
